# Evaluation of an automated ultraviolet radiation device for decontamination of *Clostridium difficile *and other healthcare-associated pathogens in hospital rooms

**DOI:** 10.1186/1471-2334-10-197

**Published:** 2010-07-08

**Authors:** Michelle M Nerandzic, Jennifer L Cadnum, Michael J Pultz, Curtis J Donskey

**Affiliations:** 1 Research Service, Louis Stokes Cleveland Veterans Affairs Medical Center, 10701 East Boulevard, Cleveland, Ohio, USA; 2 Geriatric Research, Education and Clinical Center, Cleveland Veterans Affairs Medical Center, 10701 East Boulevard, Cleveland, Ohio, USA

## Abstract

**Background:**

Environmental surfaces play an important role in transmission of healthcare-associated pathogens. There is a need for new disinfection methods that are effective against *Clostridium difficile *spores, but also safe, rapid, and automated.

**Methods:**

The Tru-D™ Rapid Room Disinfection device is a mobile, fully-automated room decontamination technology that utilizes ultraviolet-C irradiation to kill pathogens. We examined the efficacy of environmental disinfection using the Tru-D device in the laboratory and in rooms of hospitalized patients. Cultures for *C. difficile*, methicillin-resistant *Staphylococcus aureus *(MRSA), and vancomycin-resistant *Enterococcus *(VRE) were collected from commonly touched surfaces before and after use of Tru-D.

**Results:**

On inoculated surfaces, application of Tru-D at a reflected dose of 22,000 μWs/cm^2 ^for ~45 minutes consistently reduced recovery of *C. difficile *spores and MRSA by >2-3 log_10 _colony forming units (CFU)/cm^2 ^and of VRE by >3-4 log_10 _CFU/cm^2^. Similar killing of MRSA and VRE was achieved in ~20 minutes at a reflected dose of 12,000 μWs/cm^2^, but killing of *C. difficile *spores was reduced. Disinfection of hospital rooms with Tru-D reduced the frequency of positive MRSA and VRE cultures by 93% and of *C. difficile *cultures by 80%. After routine hospital cleaning of the rooms of MRSA carriers, 18% of sites under the edges of bedside tables (i.e., a frequently touched site not easily amenable to manual application of disinfectant) were contaminated with MRSA, versus 0% after Tru-D (*P *< 0.001). The system required <5 minutes to set up and did not require continuous monitoring.

**Conclusions:**

The Tru-D Rapid Room Disinfection device is a novel, automated, and efficient environmental disinfection technology that significantly reduces *C. difficile*, VRE and MRSA contamination on commonly touched hospital surfaces.

## Background

Environmental surfaces may play an important role in transmission of healthcare-associated pathogens such as *Clostridium difficile*, methicillin-resistant *Staphylococcus aureus *(MRSA), and vancomycin-resistant *Enterococcus *(VRE) [1-6]. Patients may acquire these pathogens through direct contact with contaminated surfaces or healthcare workers' hands may transmit pathogens from contaminated surfaces to susceptible patients [1-4]. Unfortunately, several recent studies have demonstrated that environmental cleaning is often suboptimal in healthcare facilities [5-8]. Interventions such as education of housekeeping staff or use of fluorescent markers to provide feedback to housekeepers may result in improved cleaning [5-8]. However, there is also a need for new disinfection methods that are effective against *Clostridium difficile *spores, but also safe, rapid, and automated.

Previous studies have demonstrated that ultraviolet-C (UV-C) irradiation kills a variety bacterial species, including *Bacillus subtilis *spores [9-15]. The mechanism of killing of microorganisms by UV-C is primarily due to inactivation of DNA and RNA through absorption of photons resulting in formation of pyrimidine dimers from thymine and cytosine [9,12,15]. The Tru-D™ Rapid Room Disinfection device (Lumalier, Memphis, TN) is a mobile, fully-automated room decontamination technology that utilizes UV-C irradiation to kill pathogens [15]. Operation of the Tru-D system involves placement of the device inside the room, closing the door, and using a wireless remote control to activate the device. Here, we examined the efficacy of environmental disinfection using the Tru-D device in the laboratory and in rooms of hospitalized patients. Housekeeping staff also incorporated the Tru-D device into their routine cleaning to assess the ease of use of the system.

## Methods

### Setting

The Cleveland Veterans Affairs Medical Center is a 202-bed acute care hospital. At the time of the study, active surveillance for MRSA carriage was performed and colonized or infected patients were placed in contact precautions. Patients with *C. difficile *infection (CDI) were placed in contact precautions until they completed treatment and diarrhea resolved. No active surveillance was performed for VRE, and VRE-colonized or infected patients were not placed in contact precautions.

### *C. difficile*, MRSA, VRE, and *Staphylococcus warneri *strains

Six *C. difficile *strains from the American Type Culture Collection (ATCC) and 2 strains cultured from patients with CDI in Cleveland were used in this study. ATCC 43593, 43602, 43603, 43596, 43599, and 43597 were standard strains representing serogroups B, K, X, C and G. VA 17 was a restriction endonuclease analysis (REA) type BI strain and VA 11 was an REA type J strain. Three MRSA strains were studied, including 2 clinical isolates with pulsed-field gel electrophoresis (PFGE) types USA300 (community-associated) and USA800, and one ATCC standard strain (43300). Three clinical VRE strains were studied including two VanA-type isolates (C37, C25) and one VanB-type isolate (C68). A single strain of *S. warneri *(ATCC 27836) was studied.

### Preparation of *C. difficile *spores

Spores were prepared by growth on Duncan and Strong agar medium as previously described [16]. Spores were stored at 4°C in sterile distilled water until use. Prior to testing, spore preps were confirmed by phase contrast microscopy and malachite green staining to be >99% dormant, bright-phase spores.

### Microbiology

For VRE, MRSA, and *C. difficile *cultures, selective media included Enterococcosel agar (Becton Dickinson, Cockeysville, MD) containing 20 μg/mL of vancomycin, CHROMagar (Becton Dickinson) containing 6 μg/mL of cefoxitin, and cycloserine-cefoxitin-brucella agar containing 0.1% taurocholic acid and lysozyme 5 mg/mL (CDBA), respectively [16]. For *S. warneri *cultures, samples were directly swabbed onto Mannitol Salt Agar (Becton Dickinson). Plates were incubated at 37°C for 48 hours. VRE and MRSA colonies with unique morphology were subjected to identification and susceptibility testing in accordance with Clinical Laboratories Standards Institute guidelines [17]. *C. difficile *was confirmed on the basis of typical odor and appearance of colonies and by a positive reaction using *C. difficile *latex agglutination (Microgen Bioproducts, Camberly, UK).

### The Tru-D device

**Figure 1:**
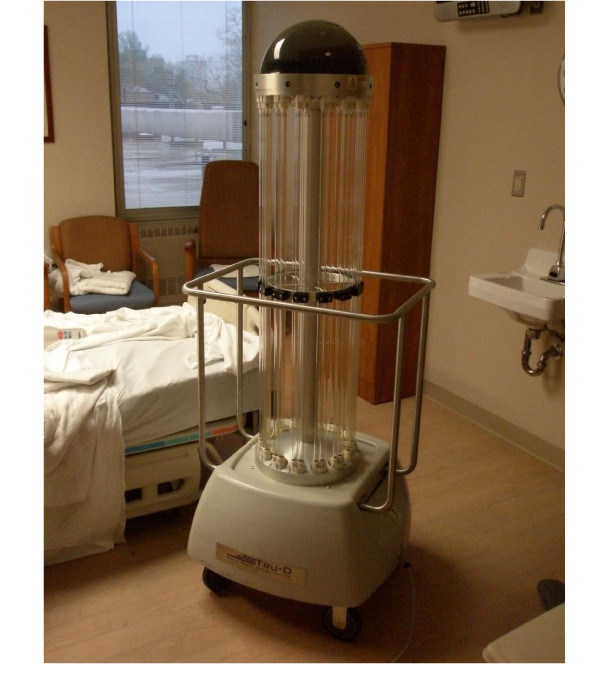
**Picture of the Tru-D device placed inside a hospital room**.

Figure 1 is a photograph of the Tru-D device. The device is 6 ft tall and measures 2 ft at the widest portion of the base. It has wheels and can easily be moved by one person. It is placed in the center of the room and commonly touched surfaces are arranged close to the device for optimal exposure to UV-C radiation (i.e. bedrails pulled up, call buttons placed on the bed, tables placed near the device). The operator exits the room, closes the door, and places a door sensor on the frame of the door. Continuous monitoring during operation is not required because the sensor triggers automatic discontinuation of the cycle if the door is opened. A handheld remote is used to select either a vegetative cycle that is effective for killing of non-spore forming organisms or a spore cycle that is effective in killing spores. The Tru-D device contains eight sensors spaced at equal distances on a ring at the top of the device. The sensors measure the amount of UV-C light reflected back to the device. The device automatically ends the cycle when the area reflecting the lowest level of UV-C back to the sensors (i.e. shaded areas in the room) has received an adequate dose [15]. UV-C radiation penetrates all areas of the room that receive light, but the highest exposure occurs for areas that are in direct line of exposure to the output of the device; areas that are not in direct line of exposure to UV-C may receive radiation that is reflected from the walls and ceiling or from other surfaces in the room.

### Reduction of pathogens inoculated onto surfaces

Initial experiments were conducted to examine the efficacy of the Tru-D device for killing *C. difficile *spores, MRSA, and VRE on laboratory bench tops. Ten μl aliquots of the organisms were inoculated onto bench top surfaces and spread to cover a 1 cm^2 ^area. Organisms were allowed to desiccate on well-lit bench tops 20 inches and 10.5 feet from the device and on a shaded surface inside a fume hood 6 feet from the Tru-D device. Previous experiments demonstrated that a reduction in vegetative organisms was observed after initial desiccation onto surfaces, however no further reduction was observed within the duration of treatment time (authors unpublished data). For each pathogen, the inoculum applied to the bench top was adjusted such that 3 to 5 log_10_CFU/cm^2 ^were recovered from the positive control specimens after desiccation (original inoculum approximately 9 log_10_CFU/mL). The surfaces were either subjected to specific reflected doses of UV-C radiation with the Tru-D device or left untreated (i.e., positive controls). Sterile swabs (BD BBL™ CultureSwab™, Becton Dickinson, Cockeysville, MD) pre-moistened with saline were applied to the surfaces and plated directly onto selective media plates and the number of colonies recovered was counted. To assess the impact of organic material on killing, similar experiments were conducted with the organisms suspended in 10 mg/ml of bovine serum albumin. Killing of the pathogens was assessed using reflected doses ranging from 5,000 to 22,000 μWs/cm^2^. The reflected doses recommended by the manufacturer are 12,000 and 22,000 μWs/cm^2 ^for killing of vegetative organisms and spores, respectively. All experiments were repeated three times.

### Disinfection in hospital rooms

The efficacy of the Tru-D device was assessed in rooms of 66 discharged patients that had not yet been cleaned by housekeeping. Organisms were not inoculated onto surfaces for these experiments. Rooms were chosen based on prior occupancy by patients on contact precaution for either MRSA or *C. difficile*. Sterile swabs pre-moistened with saline were used to collect cultures for MRSA, VRE and *C. difficile *from 4 sites (i.e., the call light, bedside table, telephone, and bed rail) before and after use of the Tru-D device at a reflected dose of 22,000-μWs/cm^2 ^(spore killing dose). For the bed rail and table, 5 × 20 cm areas were cultured before Tru-D disinfection and adjacent areas of the same size were cultured after disinfection; for the call button and telephone, half of the entire surface area (~5 × 10 cm for the call button and ~5 × 20 cm for the telephone) was cultured before and the other half after Tru-D disinfection (N = 261 total sites cultured).

An additional set of experiments was performed to determine the ability of the device to reduce levels of *C. difficile *spores on surfaces that receive only indirect exposure to UV-C radiation and that are at the farthest distance in the room from the UV-C source. Ten μl aliquots containing 5 log_10_CFU of non-toxigenic *C. difficile *spores (ATCC 43593) were inoculated onto 1 cm^2 ^areas of plastic carriers and placed either 20 inches from the device or 10 feet from the device and out of the direct line of exposure to the UV-C output (i.e., placed with the bed in between the device output and the spores). To quantify the spores, the plastic carriers were submersed in sterile deionized water and vortexed vigorously and dilutions of the suspensions were plated onto selective media. The experiment was repeated three times.

Additional experiments were performed to assess the use of the Tru-D device as an adjunct to routine housekeeping cleaning. Following discharge of 26 patients with MRSA colonization, cultures were collected for MRSA after housekeeping cleaning and again after Tru-D disinfection at a reflected dose of 12,000 μWs/cm^2 ^(vegetative killing dose). The culture sites included the call light, bedside table, telephone, and bed rail as described above. A 5 × 20 cm area of the undersurface of the edge of the bedside table was also cultured because observations indicated that it is a site that is frequently touched but not commonly cleaned and not easily amenable to manual application of disinfectant. In addition, 10 μl aliquots of *S. warneri *were inoculated and allowed to desiccate on 1 cm^2 ^areas on sites that are frequently touched but not commonly cleaned (i.e., the undersurface of the edge of the bedside table, the undersurface of the bed rail, and the lateral drawer handle of the bedside table). Sites were disinfected with bleach and allowed to air dry prior to inoculation. The inoculum applied to each site was adjusted such that 4 to 5 log_10_CFU/cm^2 ^were recovered from the positive control specimens after desiccation (original culture approximately 9 log_10_CFU/mL). Cultures were collected before and after either routine hospital cleaning or Tru-D disinfection at 12,000 μWs/cm^2^. *S. warneri *was also inoculated onto portable equipment such as pulse oximetry devices, electrocardiograph machines, and portable computers as described above and cultures were collected before and after Tru-D disinfection (N = 20). For all experiments involving *S. warneri*, negative control sites that were not inoculated with bacteria were cultured in an identical manner to evaluate the potential for environmental staphylococci to contaminate the inoculation sites. To ensure complete elimination of *S. warneri *from inoculated surfaces bleach disinfection was performed after final cultures were collected. Subsequent cultures confirmed that *S. warneri *was completely removed from all surfaces inoculated.

For a 2-week period, 2 members of the housekeeping staff were asked to incorporate use of the Tru-D device into their routine cleaning practices for rooms after patient discharge. They were asked to comment on the amount of time required to operate the device, the ease of use, the frequency of use, and any adverse effects on equipment or environmental surfaces.

### Statistical analysis

Data were analyzed using STATA 9.0 (StataCorp, College Station, TX). Continuous data were analyzed using paired *t *tests and categorical data were assessed using Fisher's exact test.

## Results

### Reduction of pathogens inoculated onto surfaces

**Figure 2:**
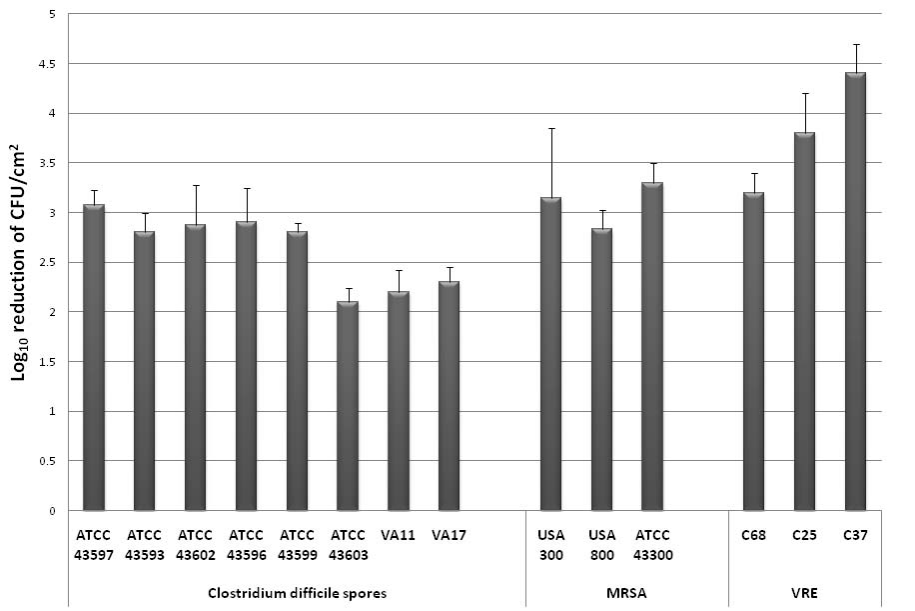
**Mean reduction (log_10_colony-forming units [CFU]/cm^2^) in recovery of multiple strains of *Clostridium difficile*, methicillin-resistant *Staphylococcus aureus *(MRSA), and vancomycin-resistant *Enterococcus *(VRE) from laboratory bench top surfaces after the use of the Tru-D device**. For each pathogen, the inoculum applied to the bench top was adjusted such that 10^3 ^to 10^5 ^CFU were recovered from the positive control specimens. The Tru-D device was operated at a reflected dose of 22,000 μWs/cm^2 ^for ~45 minutes.

Figure 2 shows the average log_10_CFU/cm^2 ^reduction of recovery of multiple strains of *C. difficile*, MRSA, and VRE from surfaces after the use of the Tru-D device. On inoculated surfaces, application of Tru-D at a reflected dose of 22,000 μWs/cm^2 ^(spore killing dose) for ~45 minutes consistently reduced recovery of *C. difficile *spores and MRSA by >2-3 log_10_CFU/cm^2 ^and VRE by >3-4 log_10_CFU/cm^2^. There were no significant differences in the levels of reduction of the pathogens when the organisms were suspended in bovine serum albumin versus PBS (*P *= 1) and when placement was 10.5 feet or 6 feet (and in a shaded location) versus 20 inches from the device (*P *≥ 0.52).

**Figure 3:**
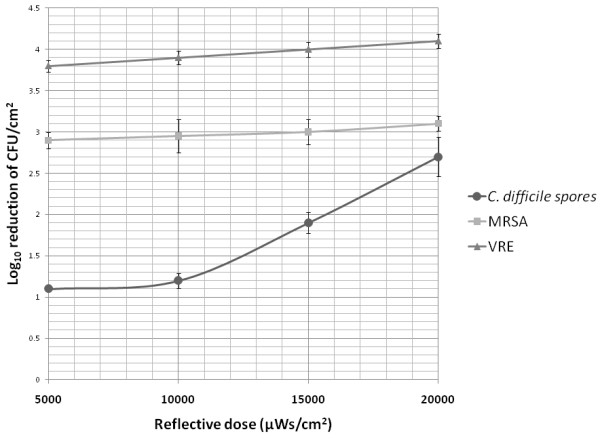
**Mean reduction (log_10_colony-forming units [CFU]/cm^2^) in recovery of 3 strains of *Clostridium difficile*, methicillin-resistant *Staphylococcus aureus *(MRSA), and vancomycin-resistant *Enterococcus *(VRE) from laboratory bench top surfaces after the use of the Tru-D device at reflected doses ranging from 5,000 to 22,000 μWs/cm^2^**.

Figure 3 shows the efficacy of Tru-D for killing of the 3 pathogens at different reflected doses. Similar levels of killing of MRSA and VRE were achieved at reflected doses ranging from 5,000 to 20,000. In contrast, killing of *C. difficile *spores increased as the reflected dose increased from 10,000 to 20,000 μWs/cm^2^.

### Disinfection in hospital rooms

**Figure 4:**
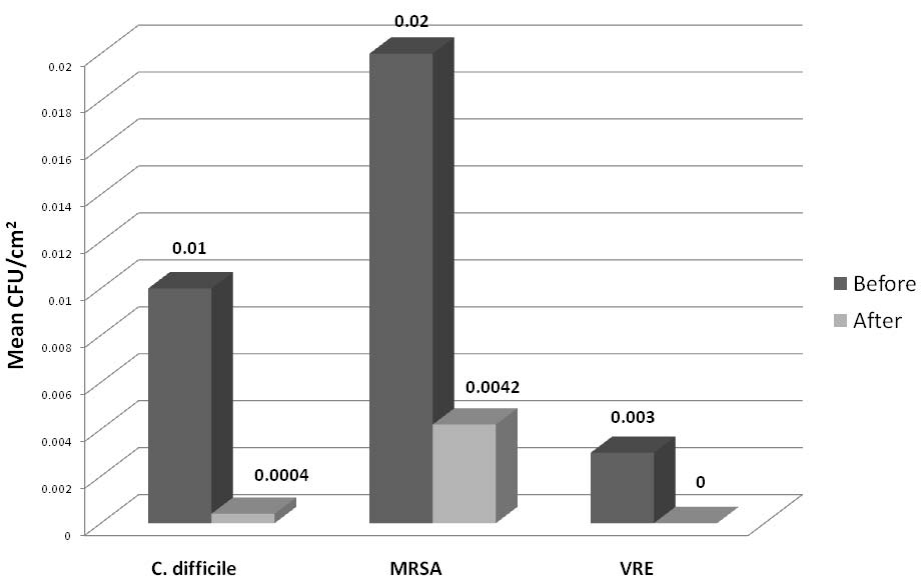
**Mean number of colony-forming units (CFU) of *Clostridium difficile*, methicillin-resistant *Staphylococcus aureus *(MRSA), and vancomycin-resistant *Enterococcus *(VRE) recovered from contaminated surfaces in hospital rooms before and after disinfection with the Tru-D device**. Two-hundred sixty-one total surfaces from 66 rooms were cultured, including call lights, bedside tables, telephones, and bed rails.

In single-patient hospital rooms, the Tru-D disinfection cycles with reflected doses of 12,000 (vegetative killing cycle) and 22,000 (spore killing cycle) μWs/cm^2 ^required about 20 and 45 minutes, respectively. For 66 isolation rooms that had not been cleaned by housekeeping (59 rooms that previously housed MRSA carriers and 7 that housed patients with *C. difficile *infection), 28 of 261 (10.7%) surfaces were positive for MRSA before disinfection at 22,000 μWs/cm^2^, and only 2 of these sites (0.8%) remained positive after Tru-D disinfection (*P *< 0.001). Seven sites (2.7%) were positive for VRE before Tru-D disinfection, versus 1 (0.38%) after disinfection (*P *= 0.07). Nine sites (3.4%) were positive for *C. difficile *before disinfection and 1 (0.38%) remained positive after Tru-D disinfection (*P *= 0.02). In addition, the mean number of CFU/cm^2 ^of each pathogen that was recovered from contaminated surfaces was significantly reduced after disinfection with the Tru-D device (*P *≤ 0.001 for each comparison) (Figure 4).

For plastic carriers inoculated with *C. difficile *spores and placed in hospital rooms, application of Tru-D significantly reduced recovery of spores at both 20 inches in direct line of UV-C exposure and at 10 feet and out of direct line of UV-C exposure (*P *≤ 0.014). However, the reduction was significantly greater at 20 inches and in direct line of exposure (2.6 log_10 _CFU/cm^2 ^versus 1 log_10 _CFU/cm^2 ^reduction, *P *< 0.009).

After housekeeping staff cleaning of 26 rooms previously occupied by MRSA carriers, no cultures of the top surfaces of the bed rails, bedside tables, call buttons, and telephones were positive. However, 18% of sites under the edges of bedside tables were contaminated with MRSA, versus 0% after Tru-D (*P *< 0.001). For 26 frequently touched but infrequently cleaned environmental sites inoculated with *S. warneri*, the number of CFUs recovered was significantly reduced after disinfection with Tru-D versus housekeeping staff (mean ± SD, 1.5 ± 1.5 versus 3.5 ± 3.7 log_10 _CFU/cm^2 ^per inoculated surface; *P *= 0.001); 50% of sites cleaned by housekeeping had high levels of contamination (i.e., > 3 log_10 _CFU/cm^2^), whereas none of the Tru-D sites had high-level contamination. Observation of housekeeping cleaning demonstrated that persistence of *S. warneri *on surfaces was due to failure to adequately apply disinfectant to the surfaces. Finally, recovery of *S. warneri *that was inoculated onto portable medical equipment (N = 20) was consistently reduced by ~2.0 log_10 _CFU/cm^2 ^after Tru-D disinfection (*P *< 0.001).

The 2 housekeepers who used the Tru-D device stated that it was easy to use and added <5 minutes to the time of room decontamination. They reported using the device up to 6 times per 8-hour work shift. An odor was noted by research and housekeeping staff members upon entering the room after the device was finished running, but it quickly dissipated after the room was reopened. There were no complaints regarding the use of the device from other staff members or patients and no apparent adverse effects on equipment or surfaces.

## Discussion

We found that the Tru-D device was effective in killing *C. difficile *spores, MRSA, and VRE inoculated onto surfaces in the laboratory and in hospital rooms. Disinfection of hospital rooms with Tru-D reduced the frequency of positive MRSA and VRE cultures by 93% and of *C. difficile *cultures by 80% on frequently touched surfaces. These findings are consistent with unpublished data from another research group that are available online [18]. We found that the device was effective in reducing contamination in sites not easily amenable to manual application of disinfectant (e.g., the undersurface of bedside tables) and on portable equipment. The housekeeping staff considered the device to be easy to use and to integrate into their routine cleaning practices. These results suggest that the Tru-D device is a promising new environmental disinfection technology that could be a useful adjunct to routine cleaning measures in healthcare facilities.

The Tru-D device has some important potential advantages over other disinfection strategies that are effective against *C. difficile *spores. Sodium hypochlorite has corrosive effects on various materials and may irritate the eyes and respiratory tracts of cleaning staff and patients, effectiveness is reduced by exposure to organic material, efficacy is dependent on correct application by housekeeping staff, and up to 10 minutes of contact time is required to achieve optimal killing of spores [19]. In contrast, use of the Tru-D device had no apparent adverse effects on surfaces or operators, efficacy was not reduced by bovine serum albumin, and the system is fully automated. Hydrogen peroxide vapor and hydrogen peroxide dry-mist have been shown to be highly effective in elimination of *C. difficile *spores [19-21]. However, these systems are relatively expensive to operate, a dedicated staff is required, and up to several hours may be required to complete room disinfection [19-21]. In contrast, after the initial purchase of the device, the cost of operating and maintaining the TRU-D device is minimal (i.e., electricity and annual bulb replacement of ~ $20 each), a dedicated staff is not essential, and the spore cycle requires only about 45 minutes.

The Tru-D device does have some potential limitations. First, it may not be feasible to use the device in circumstances where rapid turn-over of rooms is required. Second, the device cannot be used when rooms are occupied. Third, surface properties and debris may potentially inhibit lethal doses of UV-C from killing pathogens. For example, UV-C does not penetrate porous surfaces such as sheets, upholstery and curtains [15]. A previous study has shown that gross particulate matter (silica powder) significantly reduced UV-C's effectiveness in killing spores [15]. Fourth, malfunction of the sensor that triggers automatic discontinuation of the cycle if the door is opened could result in exposure of personnel or patients to UV-C. Fifth, although the Tru-D device reduces levels of pathogens on surfaces, it is not as consistently effective in eradicating contamination as hydrogen peroxide vapor [19-21]. Therefore, further studies are needed to determine if the level of reduction in contamination provided by the Tru-D device translates to reduced rates of infection. Sixth, the efficacy of the Tru-D device for killing of spores was reduced at a site that was out of direct line of exposure to UV-C and in the corner of the hospital room 10 feet from the device. Therefore, it is recommended that commonly touched surfaces (e.g., bedside table, call button, telephone) be arranged close to the device for optimal exposure to UV-C radiation. Finally, our assessment of the use of the device by housekeeping staff was limited. Additional studies are needed to evaluate whether the use of Tru-D can be easily incorporated into standard house-keeping practices.

There are also some limitations in our methodology. The use of swabs and direct plating to quantify the concentrations of organisms on surfaces is imprecise at higher concentrations. In addition, recovery of organisms from surfaces and release from swabs is less than 100%, and therefore we may not have detected lower levels of bacteria on surfaces. However, previous studies in our laboratory have indicated that the direct plating method has a limit of detection of ~10 CFU for recovery of organisms from surfaces (authors' unpublished data). In addition, the methods for processing all samples were standardized, so any limitations in the methodology were equally shared by baseline and experimental groups.

## Conclusions

The automated Tru-D Rapid Room Disinfection device significantly reduced MRSA, VRE, and *C. difficile *contamination on environmental surfaces on laboratory bench tops and in rooms of hospitalized patients. It appeared to be safe and easy to use. Further research is needed to examine whether use of the Tru-D device for environmental disinfection will reduce rates of colonization and infection with pathogens such as MRSA, VRE and *C.difficile*.

## Competing interests

The authors declare that they have no competing interests.

## Authors' contributions

MMN contributed to the study design, supervised the data collection and culture processing, participated in drafting the manuscript, and participated in editing the manuscript. JLC contributed to the study design and participated in data collection. MJP contributed to the study design and participated in data collection. CJD contributed to study design and participated in drafting and editing the manuscript. All authors read and approved the final manuscript.

## Pre-publication history

The pre-publication history for this paper can be accessed here:

http://www.biomedcentral.com/1471-2334/10/197/prepub
